# Risk of More Advanced Lesions at Hysterectomy after Initial Diagnosis of Non-Atypical Endometrial Hyperplasia in Patients with Postmenopausal Bleeding and Oral Anticoagulant Treatment

**DOI:** 10.3390/medicina57101003

**Published:** 2021-09-23

**Authors:** Adrian Carabineanu, Claudia Zaharia, Alexandru Blidisel, Razvan Ilina, Codruta Miclaus, Ovidiu Ardelean, Marius Preda, Octavian Mazilu

**Affiliations:** 1 First Department of Surgery, Second Discipline of Surgical Semiology, “Victor Babes” University of Medicine and Pharmacy, 300041 Timisoara, Romania; adriancarabi@yahoo.com (A.C.); razvanilina@yahoo.co.uk (R.I.); codrutamiclaus@yahoo.com (C.M.); ovi.ardelean@gmail.com (O.A.); predamarius2002@yahoo.com (M.P.); mazilu_o@yahoo.com (O.M.); 2 Department of Mathematics, Faculty of Mathematics and Computer Science, West University of Timisoara, 300223 Timisoara, Romania; claudia.zaharia@e-uvt.ro

**Keywords:** endometrial hyperplasia, hysterectomy, postmenopausal bleeding, oral anticoagulant treatment, endometrial lesion risk

## Abstract

*Background and Objectives*: Endometrial hyperplasia (EH) is a precursor lesion to endometrial cancer (EC), and when cellular atypia is present, in 40% of cases, they are diagnosed with EC on hysterectomy. Usually, EH is clinically manifested by uterine bleeding. In patients with oral anticoagulant therapy (OAT), the uterus is the second most common source of bleeding. The aim of the study was to show that uterine bleeding in postmenopausal patients undergoing OAT may reveal precancerous endometrial lesions with atypia, or neoplastic lesions in patients with an initial diagnosis of endometrial hyperplasia without atypia (non-atypical endometrial hyperplasia, NAEH) on dilation and curettage (D&C). We will be able to estimate the risk of a postmenopausal female patient with uterine bleeding during an OAT to have a precancerous endometrial lesion. *Materials and Methods*: The subjects of the study were 173 female patients with uterine bleeding, who have had total hysterectomy with bilateral salpingoovarectomy, of whom 99 underwent an OAT. There were 101 female patients initially diagnosed with NAEH, of which 60 did not have anticoagulant treatment (mean age 57.36 ± 6.51) and 41 had anticoagulant treatment (mean age 60.39 ± 7.35) (*p* = 0.006). From the pathology diagnosis moment, the surgery was performed at 42.09 ± 14.54 days in patients without OAT and after 35.39 ± 11.29 days in those who received such treatment (*p* = 0.724). *Results*: Initial diagnosis of NAEH established at D&C was changed at the final diagnosis after hysterectomy in EH with cellular atypia (atypical endometrial hyperplasia AEH) or EC in 18.18% of patients without OAT, and in 40.54% of patients who received this treatment. *Conclusions*: Based on a logistic regression model, it is estimated that female patients with an initial histopathological diagnosis of NAEH and who underwent OAT have, on average, 4.85 times greater odds (OR = 4.85, 95% CI 1.79–14.06) than the others of being identified postoperatively with more advanced lesions.

## 1. Introduction

Endometrial hyperplasia (EH) is a precursor lesion of EC [1,2,3]. The EH incidence is reported in 133–208 to 100,000 women per year in Western countries, 200,000 new cases per year being detected in developed countries [4,5].

Uterine bleeding is the most common clinical presentation of EH and EC in postmenopausal women [6]. The incidence of uterine bleeding in postmenopausal female patients is up to 11% [7,8,9,10].

Factors that increase the risk of malignancy in endometrial tissue include obesity, diabetes (DM) and high blood pressure (HBP) [11]. However, it is estimated that the incidence of EH is at least three times higher than of the EC [12].

EH was classified by the World Health Organization (WHO) in 2014 into two main categories, according to the presence of cytological atypia, namely EH with cellular atypia and without cellular atypia [13].

It seems that female patients with EH have a significantly increased risk of developing EC in the first four years of follow-up [11]. This risk is higher in the case of AEH; 30–40% of AEH female patients have concurrent adenocarcinoma [5,14]. There are reports showing that a quarter of AEH women diagnosed at D&C will have EC identified in the hysterectomy specimens within a few weeks after biopsy [15,16,17,18].

The first-line imaging method in the diagnosis of endometrial pathology is transvaginal ultrasound (TVUS) [19,20]. The accuracy of TVUS in diagnosing NAEH is difficult to establish. Some studies have reported a wide range of sensitivity, between 59.7–100% [21,22,23,24,25]. Hysteroscopy allows direct viewing of the endometrial cavity and allows targeted biopsy [26].

The presence of cellular atypia is the essential histological element in establishing endometrial malignancy. Outpatient endometrial biopsy does not have the accuracy expected in EH assessment and is often difficult to perform [27]. The histopathological diagnosis has a lower accuracy for endometrial lesions without atypia, and it increases when lesions with cellular atypia are present [28,29]. The risk of substaging and suboptimal treatment is not negligible [29].

The surgical treatment of endometrial pathology starting with AEH is total hysterectomy with bilateral salpingoovarectomy [30].

In patients with NAEH, hysterectomy is indicated in cases with persistent bleeding or disease progression during progestational drug treatment [31,32]. Additionally, surgical treatment is indicated as the first line in the case of contraindication of progestational treatment when there are other gynecological surgical indications or if they represent the patient’s choice [32].

Heart disease is common in patients with EC, due to its relationship to metabolic risk factors, and it causes significant morbidity [33].

The likelihood of needing oral anticoagulant or antiplatelet therapy increases with age [34]. The prevalence of OAT in the general population is reported inconsistently but is not low. Reports from Spain and Finland show shares of 1.3% and 1.6%, respectively [35,36]. Patients with heart diseases have a significant risk of thromboembolic events requiring anticoagulant treatment. The incidence of heart disease increases with age from 0.5/1000 people/year who have atrial fibrillation before the age of 50 and reaches 9.7/1000 people per year after 70 years of age [37]. Bleeding events during OAT are not uncommon [37,38]. Some studies show that the risk of bleeding associated with this treatment increases with age [39,40].

However, is bleeding a complication of anticoagulant treatment or an occult lesion? For many established yet incipient neoplastic lesions, there may be a period without clinical manifestations, but the drug anticoagulant effect produces effects on a pathologically proliferated endometrial tissue, on a vascular network-developed characteristic of a proliferative lesion.

Raposeiras et al. [41] call such an episode a “hemorrhagic stress test”. Hemorrhagic episodes during OAT highlight a significant number of unknown cancers [42].

No specific data have been established for the diagnosis of neoplastic lesions during bleeding in patients undergoing OAT [43]. Studies are reported mainly on gastrointestinal bleeding and gastrointestinal cancers [44].

The object of this study was to determine the risk of the concurrent presence of cellular atypia and endometrial malignancy in patients with NAEH and OAT, and to establish bleeding in these cases as a risk factor for a final pathology of a more advanced degree than that established in uterine curettage. Can uterine bleeding during OAT reveal a lesion with complications?

We will be able to estimate if a menopausal female patient with uterine bleeding during an OAT is subject to a precancerous or cancerous endometrial lesion when performing hysterectomy, after the histopathological exam at the bioptic curettage first showed NAEH.

## 2. Materials and Methods

A group of 175 postmenopausal female patients hospitalized and operated between 1 January 2015 and 31 December 2019, after being evaluated in outpatient gynecological consulting rooms for vaginal bleeding, have been retrospectively studied. The data were collected from medical documents issued by outpatient gynecological consulting rooms, histopathology reports based on the initial uterine evaluation, patient records from the Oncological Surgery Clinic, Victor Babes’ University of Medicine and Pharmacy Timisoara, Romania, where the surgery was performed, and the final histopathology reports, after examining the total hysterectomy specimens.

Patients were initially examined for minimal uterine bleeding. Uterine bleeding was classified as minimal if it did not give general signs of anemia, did not produce a hemodynamic imbalance, and did not require hospital treatment or blood transfusion. The bleeding was biologically assessed by performing the hemogram. A hemoglobin concentration ≥10 g/dL established a mild anemia as a result of minimal uterine bleeding [45].

The presence of obesity was also recorded and the diagnoses of high blood pressure (HBP), non-insulin-dependent type 2 diabetes mellitus (DM) and OAT were recorded from the medical records of the patients.

Patients were diagnosed with obesity according to body mass index (BMI; weight/height^2^) when BMI >30 kg/m^2^ [46].

The INR (International Normalized Ratio) determination was also performed in all patients undergoing OAT; INR determination was considered to be in the therapeutic range if the values were between 2.0–3.0 [47,48].

Imaging and histopathological evaluation was performed after clinical acknowledgement of endometrial origin of the bleeding. Diffuse endometrial thickenings ≥ 5 mm, without focal or tumor images, were diagnosed by TVUS. Endometrial histopathological evaluation by D&C was performed in all female patients in the study. When endometrial specimens were considered unsatisfactory by the pathologist, biopsy curettage was repeated associated with hysteroscopy.

The histopathological report included the evaluation of endometrial specimens according to the 2014 World Health Organization classification (WHO 2014) [49]. From these results, NAEH, AEH, and EC were registered in the study statistics. We thus considered and noted these three types of endometrial lesions as naturally evolving morphopathological stages, from NAEH to more advanced AEH lesions and later to EC.

In patients with AEH and those with EC, surgery was clearly indicated.

Female patients with NAEH on biopsy but with EC risk factors or persistent uterine bleeding after D&C were referred to the oncological surgery service for hysterectomy. The risk factors considered in this study were obesity, DM, and HBP.

In female patients included in the study, the presence of an OAT history of more than 6 months was investigated, resulting in a group without OAT (Group 1) and a group of patients with OAT history (Group 2) when requesting the gynecological consultation. The evaluation of the coagulant status was made by determining the INR at the time of the hemorrhagic event. In patients with uterine bleeding and INR values above the therapeutic limits, the lesion assessment began after the INR was within the therapeutic limits. In patients without OAT and in those with this treatment but with INR within therapeutic limits, the investigation of the cause of uterine bleeding began immediately after the hemorrhagic episode.

The time intervals from the initial histopathological evaluation by D&C and the one after hysterectomy were noted.

The criteria for including patients in the study were:

- Menopause installed for at least a year
- Light vaginal bleeding
- Imaging diffuse endometrial hyperplasia ≥ 5 mm
- Histopathological EH with or without atypia and well differentiated first stage EC
- Surgical treatment (total hysterectomy with bilateral salpingoovarectomy)

The exclusion criteria were:

- History of estrogenic or tamoxifen treatment
- Personal or family history of cancer
- Treatment of uterine bleeding with progesterone derivatives
- Imaging diagnosis of focal, polypoid or tumor lesion

Postoperative histopathological evaluation was also performed according to the WHO 2014 criteria [50].

From the anatomopathological point of view, the types of lesions detected initially at D&C, and later at the analysis of the hysterectomy pieces, were noted.

The characteristics of the initial anatomopathological lesions from D&C were investigated, as well as the consistency with the lesions later detected on the hysterectomy pieces, the involvement of risk factors for EH and EC, and the impact of the history of OAT on the anatomopathological diagnosis.

## 3. Results

The data analysis was performed using the statistical software R, version 4.0.0.

Parametric tests were used to make comparisons between groups of patients with and without OAT, namely the Student t test in the case of quantitative parameters, and respectively chi-square tests in the case of proportions. All tests were performed considering the level of significance *α* = 0.05. The existence of a relation between the number of risk factors (obesity, DM, HBP) of a patient and the degree of lesions identified postoperatively was studied using the Spearman correlation coefficient.

The impact of the present history of OAT on the risk of detecting AEH or EC lesions not detected in D&C after hysterectomy was quantified considering, for patients with an initial histopathological diagnosis of NAEH, a binary logistic regression model, including age as a covariate.

A group of 173 patients initially evaluated on an outpatient basis for minimal uterine bleeding and who underwent total hysterectomy with bilateral salpingoovarectomy between 1 January 2015 and 31 December 2019 was investigated. Of these, 99 did not undergo OAT (Group 1) and 74 had such treatment (Group 2). The clinicopathological characteristics of the 173 patients included in the study are shown in Table 1.

The mean age of the patients in the OAT group was 60.39 ± 7.35 years, while in the untreated group the mean age was 57.36 ± 6.51 years, significantly lower (*p* = 0.006).

Risk factors for EH and EC were present in close proportions in the two groups. In both groups, obesity predominated, found in 63.64% of cases in the group without a history of OAT and in 67.57% of patients in the group with OAT (*p* = 0.707). The other two risk factors, DM and HBP were identified in close proportions in the two groups, at 46.46% and44.44%, respectively, in Group 1, and at 44.59% and 45.95%, respectively in Group 2 (Table 1).

In 32 (32.32%) patients without OAT, endometrial biopsy was repeated by D&C associated with hysteroscopy, this evaluation being repeated in patients with OAT in 22 (29.72%) patients (Table 1).

The distribution of the types of endometrial lesions on initial assessment is given in Table 1. Regarding Group 1, NAEH lesions were diagnosed in 60 (60.61%) patients, while the association with atypia was found in 29 (29.29%) cases, and in the rest the presence of EC was established (10 patients, 10.1%). In Group 2, NAEH lesions were found in 41 patients (55.41%), while the association of atypia and concomitant EC was established in 22 patients (29.73%) and 11 cases (14.86%), respectively.

Our results from D&C show that there are no statistically significant differences in the distribution of histopathological types of lesions between the two groups (*p* = 0.609). Therefore, the groups are relatively homogeneous from this point of view at the time of initial diagnosis.

The final histopathological diagnosis after hysterectomy was established at 42.09 ± 14.54 days from D&C regarding Group 1 patients without a history of OAT and at 35.39 ± 11.29 days in Group 2 patients with OAT (*p* = 0.727) (Table 1). Hysterectomy specimens were analyzed histopathologically, and the consistency with initial anatomopathological diagnoses was investigated.

The types of lesions identified after hysterectomy in the two groups are shown in Table 1. Patients in Group 1 showed NAEH on hysterectomy specimens in 44 cases (44.45%) and more advanced lesions were present in 55 patients: 37 (37.37%) with AEH lesions and 18 (18.18%) with EC. In Group 2, 11 cases (14.87%) with NAEH were observed, 48 cases (64.86%) with AEH and 15 cases (20.27%) with EC.

The distribution of types of lesions after hysterectomy shows significant differences between groups (*p* = 0.0001). A much higher share of AEH can be observed in the group with anticoagulant treatment.

Regarding the concordance between the initial histological diagnosis from D&C and the diagnosis after hysterectomy, it is higher in the group without OAT: 71 patients (71.72%) show consistent results before and after hysterectomy, 25 patients (25.25%) were diagnosed after surgery as a more advanced type of lesion; in three cases (3.03%), the biopsy from the initial D&C showed more advanced lesions than the histopathological diagnosis from hysterectomy. In the group with OAT, the diagnosis after hysterectomy confirmed the initial lesion in 43 cases (58.11%), and respectively established lesions of more advanced types in 31 cases (41.89%) (Table 2).

In patients with OAT, 59 (79.73%) had INR in the therapeutic range and 15 patients (20.27%) had increased INR (Table 2). After hysterectomy, in patients with therapeutic INR the AEH-type lesions (72.88%) and EC (18.64%) predominate, while in patients with increased INR, there were 33.33% AEH type lesions and 26.67% EC type lesions. There are differences between the two categories (*p* = 0.004).

In Group 1, of the 60 patients initially identified with NAEH type lesions, for 18 (30%), AEH or EC-type lesions were found after surgery. In Group 2, out of the total of 41 patients initially identified with NAEH-type lesions, 30 (73.17%) were diagnosed after hysterectomy with more advanced types of lesions. Therefore, there is a much higher proportion of AEH and EC lesions not initially discovered in D&C in the group with a history of OAT.

For patients for whom the initial analysis found only NAEH, there were also significant differences in age: 54.70 ± 4.41 years in Group 1, compared to 61.68 ± 6.27 years in Group 2 (*p* < 0.0001). The logistic regression analysis shows that, for this category of patients, even after taking into account the potential influence of age, the history of OAT remains a significant predictor for the presence of AEH and EC endometrial lesions not discovered in D&C (Table 3).

Based on the regression model, it is estimated that in the case of patients with an initial histopathological diagnosis of NAEH, those who undergo OAT have a 4.85 times greater odds (OR = 4.85, 95% CI 1.79–14.06) than the others, to display changes resulting from more advanced lesions (AEH or EC) after hysterectomy. The obtained model is significant (likelihood ratio test, *p* < 0.00001) and adequate (Hosmer–Lemeshow concordance test, *p* = 0.243).

## 4. Discussion

The detection of atypical endometrial lesions is a histological challenge. Therefore, there is a risk of underdiagnosis and, consequently, of undertreatment, which is not negligible in published reports [49].

Attempts to improve the early detection of cases of atypical or neoplastic endometrial lesions are beneficial, helping to reduce situations of unexpected diagnosis of precancerous lesions.

Unlike in cervical cancer, a pathology in which performing the Papanicolau test cytologically helped to diagnose the early stages of cancer in the early detection of precancerous or EC lesions, precise methodologies have not yet been established [51,52,53].

The female genital tract is the location with the most frequent episodes of bleeding in the presence of pathologies predisposing to hemorrhage in patients undergoing OAT [54]. Bleeding is also more common in patients with latent pathology [55].

A recent study established a three-fold higher risk of establishing a new cancer diagnosis in patients with non-major bleeding during an OAT [41].

This study is similar to that reported by Eikelboom et al. [54] from the COMPASS trial (Cardiovascular Outcomes for People Using Anticoagulation Strategies), which concluded that 69% of newly detected cancers were found in patients with non-major bleeding.

All patients in our research showed light bleeding without the need for a blood transfusion. In our study, the mean intervals between D&C diagnosis and the final diagnosis after hysterectomy were 42.09 ± 14.54 days for Group 1 and 35.39 ± 11.29 days for Group 2 with OAT. Data were reported in the interval between bleeding and the pathological diagnosis after hysterectomy, resulting in a 6-month interval with increased risk for EC detection [41,56].

It is common to establish the origin of bleeding as asymptomatic lesions, when the hemorrhagic event occurs in a patient with OAT, and INR within therapeutic limits [55,57]. In our research, in the group with OAT and INR within therapeutic limits, the bleeding was caused by an AEH or neoplastic lesion in 49.15% of cases.

Research showed that the risk of bleeding associated with OAT is associated with INR values [58,59]. Accordingly, with the increase in INR, the risk of bleeding also increases.

We found that, when the INR showed values above the therapeutic ones, 11 patients (73.33%) were diagnosed with D&C with NAEH, and 4 (26.66%) had EC lesions. The initial histopathological diagnosis was changed in 5 patients out of the 11 with NAEH, representing 45.45%, in which AEH lesions were detected at hysterectomy.

The proportion of patients with increased INR with discordant results at the two histopathological analyses, D&C and hysterectomy, is 33.33%, lower than 44.07%, the proportion of patients with discordant results with normal INR; however, the difference between the groups is not statistically significant (*p* = 0.646). Therefore, the idea that an increased INR would be associated with a higher potential for discordant results in the two analyses is not supported.

The ACOG (American College of Obstetricians and Gynecologists) recommends TVUS as being appropriate in the initial assessment of postmenopausal patients with initial endometrial bleeding, setting an endometrial thickening of up to 4mm as the normal ultrasound limit [53,60,61,62,63,64,65].

Ultrasound thickness of the endometrium has been considered in most studies as an indicator of endometrial pathology, but recent studies have shown that an abnormal endometrial line morphology in TVUS would be a better predictor [66]. In our research, the ultrasound evaluation included patients with an endometrial thickness over 5 mm, with irregular morphology at the endometrial line, but without evidence of polypoid or focal lesions.

Postmenopausal women with uterine bleeding and endometrial thickness ≤ 4 mm are less likely to have malignancy [67,68].

In ordinary medical practice, the histopathological diagnosis of endometrial pathology, EH, is also established by analyzing histological samples taken by D&C. Endometrial D&C is considered appropriate for endometrial pathological diagnosis [69,70]. D&C can help detect EC preoperatively but is not accurate enough to rule out malignancy [71,72,73,74,75,76].

We considered D&C a sufficient criterion for including patients in the study. The research was performed on patients with EH, excluding those with focal hyperplasia or an endometrial polyp, when a targeted biopsy procedure was more appropriate. Providing satisfactory tissue to the pathologist for diagnosis was the necessary common criterion of preoperative histopathological diagnostic procedures.

Some authors recommend hysteroscopy in all patients investigated for endometrial pathology, while other studies recommend re-biopsy in the presence of risk factors in the case of insufficient initial tissue specimens for diagnosis [32,77,78].

In our research, hysteroscopy was not used constantly, but only in cases where samples collected from D&C were considered insufficient for diagnosis by the pathologist. Endometrial re-biopsy by D&C associated with hysteroscopy was performed in 32.32% of patients without OAT and in 29.72% of patients with OAT (Table 1). These results are consistent with other reports that established different proportions of insufficient tissue cases for histological examination after D&C, in 17–31.7% of cases [79,80].

There is no systematic correlation between hysteroscopic features and the diagnosis of EH [33]. On the other hand, a meta-analysis that included patients who underwent hysteroscopy found a significant degree of dissemination with neoplastic endometrial cells in the peritoneal cavity in patients with EC [81].

Regarding the classification of endometrial lesions, we classified the presentations in the morpho-pathological bulletins into three types: NAEH, which includes all histological changes of hyperplasia in endometrial specimens in which no cell atypia were detected, AEH when cellular atypia was associated with hyperplasia, and EC (well-differentiated endometrial adenocarcinoma).

Although the 1994 WHO classification (WHO94) of EH is the most widely used one by pathologists in histopathological bulletins, we included endometrial lesions based on the WHO2014 classification, recommended as the best classification and management scheme for EH [13,50,82].

We did not perform more advanced imaging investigations of the patients in the study. We considered that, at this stage of endometrial pathology, it does not provide significant data. Recent studies show that magnetic resonance imaging (MRI) is not considered an effective way to identify invasive lesions in EH cases and can give false positive results in 46% of cases [12,50,83].

Age has been reported to be strongly correlated with the risk of EC, and also the risk of diagnosis of occult cancer is strongly related to age [84,85].

In our research, there are significant differences between the groups in terms of average age, which is higher in the OAT group, 60.39 ± 7.35 years compared to 57.36 ± 6.51 years in Group 1 without OAT (*p* = 0.006). Lee et al. [86] published a study in which patients ≥ 51 years of age with NAEH had an approximately 50% risk of occult AEH or EC lesions.

The fact that there are differences between groups in terms of age can make this parameter, from a statistical point of view, a so-called “confounding variable”—in this case, the diagnosis of AEH and EC more frequently after hysterectomy is somehow related to age, slightly more advanced, and not dependent on the application of the anticoagulant treatment. One way to solve the problem was to use the logistic regression model, which shows that even after adjusting with age, the treatment is the one that influences the preseance of the diagnosis of AEH and EC.

Risk factors for EC are age, endometrial thickening, obesity, DM, and HBP [87].

In our study, patients with NAEH underwent total hysterectomy if they had risk factors for EC, or if bleeding persisted after D&C. Recent studies have shown that patients with initial histology of NAEH, who had recurrent uterine bleeding, had early endometrial neoplasia on further histological evaluation [11].

The analysis of the frequency of risk factors shows that they do not differ significantly between the two groups.

Practically, the proportion of obese patients between the group with anticoagulant history (67.57%) and the one without anticoagulant treatment (63.64%, *p* = 0.707) does not significantly differ, nor does the proportion of patients with diabetes between Group 1 (44.59%) and Group 2 (46.46%, *p* = 0.928). Additionally, the proportion of patients with HBP does not differ significantly between the group with OAT (45.95%) and the one without a history of OAT (44.44%, *p* = 0.967).

Regarding the association between risk factors for EH and EC and the type of lesions on hysterectomy specimens, a positive, significant correlation was identified between the degree of lesions identified postoperatively and the number of risk factors a patient is predisposed to (Spearman *ρ* = 0.434 in Group 1, *ρ* = 0.204 in Group 2).

In hysterectomy specimens, EC was established in 18 cases (18.18%) of patients with uterine bleeding without OAT (Group 1). In one patient in this group, the initial diagnosis of NAEH was changed in the final diagnosis of EC, representing 1.6% of patients. Hui et al. [88] documented the change in initial diagnosis of NAEH to EC in 4.2% of cases. Similar research reported the same change in the initial diagnosis of NAEH after hysterectomy, between 1.1–13.7% of cases [88,89,90,91,92,93].

A study reported that 13.7% of patients diagnosed with D&C with NAEH had a final diagnosis of EC in hysterectomy specimens [89]. Additionally, the same research documented the same change in histological diagnosis from AEH to EC in 66.6% of patients. In our research of the 29 patients in Group 1 initially diagnosed with AEH, in seven (24.13%) of them, EC was detected in hysterectomy specimens.

In our study, we found the detection of cellular atypia in 17 (28.33%) patients in Group 1 who were initially diagnosed with NAEH. In other studies, this diagnostic change was reported in 1.1–50% of cases with an initial diagnosis of NAEH [49,73,74,75].

Hui et al. [88] reported that patients with NAEH on the initial histological evaluation were diagnosed in hysterectomy specimens with cell atypia in 18.8% of cases.

In another study comprising 281 patients with NAEH, 12.1% of them were diagnosed with occult AEH lesions [86].

The proportion of patients for whom the postoperative outcome indicates lesions in a more advanced type than initially assessed is significantly higher in the OAT group than in the untreated group (*p* = 0.032). It should be noted that most of the changes in the histopathological lesion diagnosis from the initial one to post- hysterectomy in a superior lesion type are of the NAEH → AEH type (Table 2).

We studied the concordance of cumulative diagnoses of patients with precancerous histopathological lesions AEH and EC. The number of initial anatomopathological diagnoses from D&C of AEH and EC almost doubled from 33 to 63 cases in the OAT group, with a less pronounced trend in patients in the group without this treatment, from 39 cases initially to 55 cases on hysterectomy pieces. Statistically, studying the involvement of OAT in this trend showed that the treatment did not influence the D&C diagnosis of AEH and EC-type endometrial lesions (*p* = 0.596).

When we investigated this behavior of the histopathological diagnosis on the hysterectomy pieces, it turned out that OAT influenced the diagnosis of AEH and EC lesions (*p* < 0.0001).

It is known that early diagnosis of gynecological neoplastic lesions helps to perform early treatment and a better prognosis [94]. There is the question of whether patients with uterine bleeding and OAT are, in fact, asymptomatic patients appearing in study articles, especially in the idea of screening and detecting early precancerous lesions. Kimura et al. [95] demonstrated better survival of asymptomatic patients compared to those diagnosed after the onset of uterine bleeding. In asymptomatic patients, there are also reports of endometrial thickening > 11 mm as the limit for endometrial biopsy [96].

In our research we included only well-differentiated proliferative lesions due to the complexity of the problem. There are published studies that show the different biological and clinical features of proliferative endometrial lesions. According to them, well and slightly differentiated lesions have a lower predisposition to clinical uterine bleeding compared to poorly differentiated ones [97].

The concern about identifying risk factors for these situations is present in the studies of many authors who try to stratify risk factors. Aside from the traditional risk factors of endometrial neoplasia, there are studies on other factors, such as surgical indication and postmenopausal bleeding [98]. Research has been published in which 7.92% of cases were diagnosed with occult endometrial cancer lesions in patients with postmenopausal bleeding hysterectomized for benign conditions [98].

### Study Limitations

There are some limitations to this study. First, our research covers the patient population and clinical practice in a single medical clinic.

Secondly, our study was performed on retrospective data to identify hysterectomies performed for surgical indications based on cell atypia, incipient neoplastic lesions or NAEH with recurrent bleeding or risk factors. Conclusions have been reported that retrospective studies generally report a higher prevalence of occult uterine cancer than prospective studies [99,100].

Moreover, the patients studied represent a population selected by the very indication of hospitalization for surgery.

The presence of risk factors in the operability criteria of patients with NAEH may influence the results. Additionally, the research refers to a group of “surgical” patients due to the surgical specificity of the documentation source. However, considering that surgeries for this pathology are given a lower or higher degree of prevention, we believe that the results obtained in this study strengthen the benefit we pursue in the treatment of this pathology.

There are differences between the two categories in the group with a history of OAT with therapeutic INR and increased INR in terms of the frequency of more serious lesions and the frequency of discordant lesions. These should be interpreted with caution due to the small volume of data, and they do not support an association between an increased INR and a higher frequency of more severe lesions or a higher frequency of discordant lesions between the initial histopathological examination and the one after hysterectomy.

## 5. Conclusions

Uterine bleeding during OAT may facilitate the determination of the risk of precancerous endometrial lesions.

The prediction model in our study reflects a preliminary step in this direction. For example, for patients of different ages, comorbidities, and surgical indications, one may try to predict the likelihood of having unexpected precancerous endometrial lesions.

Determining the true endometrial lesion situation as accurately as possible subjects many practitioners to questions and trials. As this study shows, a history of OAT can cause bleeding from an otherwise asymptomatic early lesion. On the other hand, endometrial vaginal bleeding caused by anticoagulants may be independent of the lesion. After all, an immediate request for a medical consultation by the patient concerned about bleeding is a gain in itself.

Our research has shown the ability of hypocoagulation to complicate an asymptomatic endometrial lesion early in the absence of anticoagulant effects or well-differentiated lesions that naturally have a lower ability to cause uterine bleeding in postmenopause. Based on this model, we can estimate that in the case of patients who initially have only NAEH-type changes, those who perform OAT have a 4.85 times higher risk than those who do not follow such a treatment, with evidence of histopathological changes at a more advanced stage, AEH or EC, on the hysterectomy pieces.

The existence of OAT can be used to stratify the risk and to discuss the surgery; this can help the preoperative assessment of the risks of these patients, finally improving the prognosis of these patients.

## Figures and Tables

**Table 1:** Characteristics of patients in the two study groups.

Patients with Uterine Bleeding*N* = 173		Group 1 (Without Anticoagulant Treatment)*N* = 99	Group 2 (With Anticoagulant Treatment)*N* = 74	*p*
Age (years)		57.36 ± 6.51 *	60.39 ± 7.35 *	0.006 **
Risk factors				
Obesity *N* (%)		63 (63.64)	50 (67.57)	0.707 ***
Diabetes mellitus *N* (%)		46 (46.46)	33 (44.59)	0.928 ***
High blood pressure *N* (%)		44 (44.44)	34 (45.95)	0.967 **
Repeated biopsy *N* (%)		32 (32.32)	22 (29.72)	
Histopathological diagnosis on D&C *N* (%)	NAEHAEHEC	60 (60.61)29 (29.29)10 (10.1)	41 (55.41)22 (29.73)11 (14.86)	0.609 ***
Histopathological diagnosis on hysterectomy *N* (%)	NAEHAEHEC	44 (44.45)37 (37.37)18 (18.18)	11 (14.87)48 (64.86)15 (20.27)	0.0001 ***
Diagnosis interval D&C-hysterectomy (days)		42.09 ± 14.5 *	35.39±11.29 *	0.727 **
Increased INR *N* (%)Therapeutic INR *N* (%)		--	15 (20.27)59 (79.72)	

* mean ± standard deviation, ** *t* test, *** chi square test, *N* = number of patients, NAEH = non-atypical endometrial hyperplasia, AEH = cellular atypia endometrial hyperplasia, EC = endometrial cancer, D&C = dilation and curettage, INR = International Normalized Ratio.

**Table 2:** Initial diagnosis (from D&C) and final diagnosis (from hysterectomy) in the two groups of patients.

Group	D&C-Hysterectomy								Total
	NAEH-NAEH	NAEH-AEH	NAEH-EC	AEH-AEH	AEH-EC	EC-EC	EC-AEH	AEH-NAEH	
1 *N* (%)	42 (42.42)	17 (17.17)	1 (1.01)	20 (20.2)	7 (7.07)	9 (9.09)	1 (1.01)	2 (2.02)	99
2 *N* (%)	11 (14.86)	27 (36.48)	3 (4.05)	21 (28.37)	1 (1.35)	11 (14.86)	-	-	74
Therapeutic INR *N* (%)	5 (8.4)	22 (37.28)	3 (5.08)	21 (35.59)	1 (1.69)	7 (11.86)	-	-	59
Increased INR *N* (%)	6 (40.00)	5 (33.33)	-	-	-	4 (26.66)	-	-	15

Group 1—patients without oral anticoagulant treatment, Group 2—patients with oral anticoagulant treatment, *N* = number, NAEH = non-atypical endometrial hyperplasia, AEH = cellular atypia endometrial hyperplasia, EC = endometrial cancer, D&C = dilation and curettage, INR = International Normalized Ratio.

**Table 3:** Coefficients of the logistic regression model that characterize the probability that a patient with NAEH at D&C will be diagnosed with more advanced lesions after hysterectomy, in relation with age and history of OAT.

Variable	Coefficient	*p*	OR	95% CI
Group 2 (Anticoagulant treatment)	1.58	0.002	4.85	1.79–14.06
Age	0.04	0.331	1.04	0.96–1.14

Group 1—group with patients without anticoagulant treatment, Group 2—group with patients with anticoagulant treatment; the reference level of the qualitative predictor is considered to belong to Group 1.

## Data Availability

The datasets used and/or analyzed during the present study are available from the first author on reasonable request.

## References

[1] Horn L.C., Schnurrbusch U., Bilek K., Hentschel B., Einenkel J. (2004). Risk of Progression in Complex and Atypical Endometrial Hyperplasia: Clinicopathologic Analysis in Cases with and without Progestogen Treatment. Int. J. Gynecol. Cancer.

[2] Daud S., Jalil S.S., Griffin M., Ewies A.A. (2011). Endometrial Hyperplasia-The Dilemma of Management Remains: A Retrospective Observational Study of 280 Women. Eur. J. Obstet. Gynecol. Reprod. Biol..

[3] Kurman R.J., Kaminski P.F., Norris H.J. (1985). The Behavior of Endometrial Hyperplasia. A Long-Term Study of “Untreated” Hyperplasia in 170 Patients. Cancer.

[4] Ozdegirmenci O., Kayikcioglu F., Bozkurt U., Akgul M.A., Haberal A. (2011). Comparison of the Efficacy of Three Progestins in the Treatment of Simple Endometrial Hyperplasia without Atypia. Gynecol. Obstet. Investig..

[5] Reed S.D., Newton K.M., Clinton W.L., Epplein M., Garcia R., Allison K., Voigt L.F., Weiss N.S. (2009). Incidence of Endometrial Hyperplasia. Am. J. Obstet. Gynecol..

[6] Sorosky J.I. (2012). Endometrial Cancer. Obstet. Gynecol..

[7] Astrup K., Olivarius N.d.F. (2004). Frequency of Spontaneously Occurring Postmenopausal Bleeding in the General Population. Acta Obstet. Gynecol. Scand..

[8] Rossouw J.E., Anderson G.L., Prentice R.L., LaCroix A.Z., Kooperberg C., Stefanick M.L., Jackson R., Beresford S.A., Howard B.V., Johnson K.C. (2002). Writing Group for the Women’s Health Initiative Investigators. Risks and Benefits of Estrogen Plus Progestin in Healthy Post-Menopausal Women: Principal Results from the Women’s Health Initiative Randomized Controlled Trial. JAMA.

[9] Smith-Bindman R., Weiss E., Feldstein V. (2004). How Thick Is Too Thick? When Endometrial Thickness Should Prompt Biopsy in Postmenopausal Women without Vaginal Bleeding. Ultrasound Obstet. Gynecol..

[10] Mirkin S., Archer D.F., Taylor H.S., Pickar J.H., Komm B.S. (2014). Differential Effects of Menopausal Therapies on the Endometrium. Menopause.

[11] Visser N.C., Sparidaens E.M., van den Brink J.W., Breijer M.C., Boss E.A., Veersema S., Siebers A.G., Bulten J., Pijnenborg J.M., Bekkers R.L. (2016). Long-Term Risk of Endometrial Cancer following Postmenopausal Bleeding and Reassuring Endometrial Biopsy. Acta Obstet. Gynecol. Scand..

[12] Natarajan P., Vinturache A., Hutson R. (2020). The Value of MRI in Management of Endometrial Hyperplasia with Atypia. World J. Surg. Onc..

[13] Emons G., Beckmann M.W., Schmidt D., Mallmann P. (2015). Uterus commission of the Gynecological Oncology Working Group (AGO) New WHO Classification of Endometrial Hyperplasias. Geburtshilfe Frauenheilkd.

[14] Montgomery B.E., Daum G.S., Dunton C.J. (2004). Endometrial Hyperplasia: A Review. Obstet. Gynecol. Surv..

[15] Archer D., McIntyre-Seltman K., Wilborn W., Dowling E., Conce F., Creasy G. (1991). Endometrial Morphology in Asymptomatic Postmenopausal Women. Am. J. Obstet. Gynecol..

[16] Gull B., Karlsson B., Milsom I., Wikland M., Granberg S. (1996). Transvaginal Sonography of the Endometrium in a Representative Sample of Postmenopausal Women. Ultrasound Obstet. Gynecol..

[17] Koss L.G., Schreiber K., Oberlander S.G., Moussouris H.F., Lesser M. (1984). Detection of Endometrial Carcinoma and Hyperplasia in Asymptomatic Women. Obstet. Gynecol..

[18] Reid R., Roberts F., MacDuff E. (2011). Female Genital System and Breast in Pathology Illustrated. Pathology Illustrated E-Book.

[19] Timmermans A., Opmeer B.C., Khan K.S., Bachmann L.M., Epstein E., Clark T.J., Gupta J.K., Bakour S.H., van den Bosch T., van Doorn H.C. (2010). Endometrial Thickness Measurement for Detecting Endometrial Cancer in Women with Postmenopausal Bleeding. Obstet. Gynecol..

[20] Gupta J.K., Chien P.F.W., Voit D. (2002). Ultrasonographic Endometrial Thickness for Diagnosing Endometrial Pathology in Women with Postmenopausal Bleeding: A Meta-Analysis. Acta Obstet. Gynecol. Scand..

[21] Ryu J.-A., Kim B., Lee J., Kim S., Lee S.H. (2004). Comparison of Transvaginal Ultrasonography with Hysterosonography as a Screening Method in Patients with Abnormal Uterine Bleeding. Korean J. Radiol..

[22] Shokouhi B. (2015). Role of Transvaginal Ultrasonography in Diagnosing Endometrial Hyperplasia in Pre- and Post-Menopause Women. Niger. Med. J..

[23] Gambacciani M., Monteleone P., Ciaponi M., Sacco A., Genazzani A. (2004). Clinical Usefulness of Endometrial Screening by Ultrasound in Asymptomatic Postmenopausal Women. Maturitas.

[24] Koss L.G., Schreiber K., Oberlander S.G., Moukhtar M., Levine H.S., Moussouris H.F. (1981). Screening of Asymptomatic Women for Endometrial Cancer. CA A Cancer J. Clin..

[25] Vuento M.H., Pirhonen J.P., Makinen J.I., Tyrkko J.E., Laippala P.J., Gronroos M., Salmi T.A. (1999). Screening for Endometrial Cancer in Asymptomatic Postmenopausal Women with Conventional and Colour Doppler Sonography. BJOG Int. J. Obstet. Gynaecol..

[26] Bakour S.H., Khan K.S., Gupta J.K. (2000). Transvaginal Ultrasonography and Endometrial Histology in Peri-And Post-Menopausal Women on Hormone Replacement Therapy. BJOG Int. J. Obstet. Gynaecol..

[27] Clark T.J., Mann C.H., Shah N., Khan K.S., Song F., Gupta J.K. (2001). Accuracy of Outpatient Endometrial Biopsy in the Diagnosis of Endometrial Hyperplasia. Acta Obstet. Gynecol. Scand..

[28] Zaino R., Kauderer J., Trimble C.L., Silverberg S.G., Curtin J.P., Lim P.C., Gallup D.G. (2006). Reproducibility of the Diagnosis of Atypical Endometrial Hyperplasia: A Gynecologic Oncology Group Study. Cancer.

[29] Colombo N., Creutzberg C., Amant F., Bosse T., Martín A.G., Ledermann J., Marth C., A Nout R., Querleu D., Mirza M.R. (2016). ESMO-ESGO-ESTRO Consensus Conference on Endometrial Cancer: Diagnosis, Treatment and Follow-Up. Int. J. Gynecol. Cancer.

[30] Iversen M.L., Dueholm M. (2018). Complex Non Atypical Hyperplasia and the Subsequent Risk of Carcinoma, Atypia and Hysterectomy during the Following 9–14 Years. Eur. J. Obstet. Gynecol. Reprod. Biol..

[31] Twu N.F., Chen S.S. (2000). Five-Year Follow-Up of Patients with Recurrent Postmenopausal Bleeding. Zhonghua Yi Xue Za Zhi.

[32] Royal College of Obstetricians & Gynaecologists Management of Endometrial Hyperplasia. https://www.rcog.org.uk/globalassets/documents/guidelines/green-top-guidelines/gtg_67_endometrial_hyperplasia.pdf.

[33] Ward K.K., Shah N.R., Saenz C.C., McHale M.T., Alvarez E.A., Plaxe S.C. (2012). Cardiovascular Disease Is the Leading Cause of Death among Endometrial Cancer Patients. Gynecol. Oncol..

[34] Navarro J.L., Cesar J.M., Fernández M.A., Fontcuberta J., Reverter J.C., Gol-Freixa J. (2007). Morbidity and Mortality in Patients Treated with Oral Anticoagulants. Rev. Esp. Cardiol..

[35] Virjo I., Mäkelä K., Aho J., Kalliola P., Kurunmäki H., Uusitalo L., Valli M., Ylinen S. (2010). Who Receives Anticoagulant Treatment with Warfarin and Why? A Population-Based Study in Finland. Scand. J. Prim. Heal. Care.

[36] Krahn A.D., Manfreda J., Tate R.B., Mathewson F.A., Cuddy T.E. (1995). The Natural History of Atrial Fibrillation: Incidence, Risk Factors, and Prognosis in the Manitoba Follow-Up Study. Am. J. Med..

[37] Sherwood M.W., Nessel C.C., Hellkamp A.S., Mahaffey K.W., Piccini J.P., Suh E.-Y., Becker R.C., Singer D.E., Halperin J.L., Hankey G. (2015). Gastrointestinal Bleeding in Patients with Atrial Fibrillation Treated with Rivaroxaban or Warfarin. J. Am. Coll. Cardiol..

[38] O’Brien E.C., Simon D.N., Thomas L.E., Hylek E.M., Gersh B.J., Ansell J.E., Kowey P.R., Mahaffey K.W., Chang P., Fonarow G.C. (2015). The ORBIT Bleeding Score: A Simple Bedside Score to Assess Bleeding Risk in Atrial Fibrillation. Eur. Hear. J..

[39] Hutten B.A., Lensing A.W.A., Kraaijenhagen R.A., Prins M.H. (1999). Safety of Treatment with Oral Anticoagulants in the Elderly. Drugs Aging.

[40] Van der Meer F.J., Rosendaal F.R., Vandenbroucke J.P., Briët E. (1996). Assessment of a Bleeding Risk Index in Two Cohorts of Patients Treated with Oral Anticoagulants. Thromb. Haemost..

[41] Raposeiras S.R., Abu Assi E., Pardal C.B., Fernandez M.C., Pousa I.M., Paz R.C., Parada J.A., Montenegro M.R., Miu M.M., Prieto S.B. (2020). New Cancer Diagnosis after Bleeding in Anticoagulated Patients with Atrial Fibrillation. J. Am. Hear. Assoc..

[42] Fang M.C., Chang Y., Hylek E.M., Rosand J., Greenberg S.M., Go A.S., Singer D.E. (2004). Advanced Age, Anticoagulation Intensity, and Risk for Intracranial Hemorrhage Among Patients Taking Warfarin for Atrial Fibrillation. Ann. Intern. Med..

[43] Nørgaard M., Veres K., Ording A.G., Djurhuus J.C., Jensen J.B., Sørensen H.T. (2018). Evaluation of Hospital-Based Hematuria Diagnosis and Subsequent Cancer Risk Among Adults in Denmark. JAMA Netw. Open.

[44] Rasmussen P.V., Dalgaard F., Gislason G.H., Brandes A., Johnsen S.P., Grove E.L., Torp-Pedersen C., Dybro L., Harboe L., Münster A.-M.B. (2020). Gastrointestinal Bleeding and the Risk of Colorectal Cancer in Anticoagulated Patients with Atrial Fibrillation. Eur. Hear. J..

[45] Beutler E., Waalen J. (2006). The Definition of Anemia: What Is the Lower Limit of Normal of the Blood Hemoglobin Concentration?. Blood.

[46] Global BMI Mortality Collaboration (2016). Body-Mass Index and All-Cause Mortality: Individual-Participant-Data Meta-Analysis of 239 Prospective Studies in Four Continents. Lancet.

[47] Wieland E., Shipkova M. (2019). Pharmacokinetic and Pharmacodynamic Drug Monitoring of Direct-Acting Oral Anticoagulants: Where Do We Stand?. Ther. Drug Monit..

[48] Pourafkari L., Baghbani-Oskouei A., Savadi-Oskouei S., Ghaffari S., Parizad R., Tajlil A., Nader N.D. (2019). Prediction Model for Significant Bleeding in Patients with Supratherapeutic International Normalized Ratio after Oral Administration of Warfarin. Clin. Drug Investig..

[49] Armstrong A.J., Hurd W.W., Elguero S., Barker N.M., Zanotti K.M. (2012). Diagnosis and Management of Endometrial Hyperplasia. J. Minim. Invasive Gynecol..

[50] Allison K.H., Reed S.D., Voigt L.F. (2008). Diagnosis Endometrial Hyperplasia: Why Is It So Difficult to Agree?. Am. J. Surg. Pathol..

[51] Vitale S.G. (2020). The Biopsy Snake Grasper Sec. VITALE: A New Tool for Office Hysteroscopy. J. Minim. Invasive Gynecol..

[52] (2009). ACOG Committee Opinion No. 440: The Role of Transvaginal Ultrasonography in the Evaluation of Postmenopausal Bleeding. Obstet. Gynecol..

[53] Goldstein R.B., Bree R.L., Benson C.B., Benacerraf B.R., Bloss J.D., Carlos R., Fleischer A.C., Goldstein S.R., Hunt R.B., Kurman R.J. (2001). Evaluation of the Woman with Postmenopausal Bleeding: Society of Radiologists in Ultrasound-Sponsored Consensus Conference Statement. J. Ultrasound Med..

[54] Eikelboom J.W., Connolly S.J., Bosch J., Shestakovska O., Aboyans V., Alings M., Anand S.S., Avezum A., Berkowitz S.D., Bhatt D.L. (2019). Bleeding and New Cancer Diagnosis in Patients with Atherosclerosis. Circulation.

[55] Lavítola P.D.L., Spina G.S., Sampaio R.O., Tarasoutchi F., Grinberg M. (2009). Sangramento durante a Anticoagulação Oral: Alerta Sobre um Mal Maior. Arq. Bras. Cardiol..

[56] Clemens A., Strack A., Noack H., Konstantinides S., Brueckmann M., Lip G.Y.H. (2014). Anticoagulant-Related Gastrointestinal Bleeding-Could This Facilitate Early Detection of Benign or Malignant Gastrointestinal Lesions?. Ann. Med..

[57] Ansell J., Hirsh J., Dalen J., Bussey H., Anderson D., Poller L., Jacobson A., Deykin D., Matchar D. (2001). Managing Oral Anticoagulant Therapy. Chest.

[58] Hylek E.M., Skates S.J., Sheehan M.A., Singer D.E. (1996). An Analysis of the Lowest Effective Intensity of Prophylactic Anticoagulation for Patients with Nonrheumatic Atrial Fibrillation. N. Engl. J. Med..

[59] Levine M.N., Raskob G., Landefeld S., Kearon C. (2001). Hemorrhagic Complications of Anticoagulant Treatment. Chest.

[60] Rakha E., Wong S.C., Soomro I., Chaudry Z., Sharma A., Deen S., Chan S., Abu J., Nunns D., Williamson K. (2012). Clinical Outcome of Atypical Endometrial Hyperplasia Diagnosed on an Endometrial Biopsy. Am. J. Surg. Pathol..

[61] Saso S., Chatterjee J., Georgiou E., Ditri A.M., Smith J.R., Ghaem-Maghami S. (2011). Endometrial Cancer. BMJ.

[62] Braun M.M., Overbeek-Wager E., Grumbo R.J. (2016). Diagnosis and Management of Endometrial Cancer. Am. Fam. Physician.

[63] (2013). ACOG Committee Opinion No. 557. Obstet. Gynecol..

[64] (2018). ACOG Committee Opinion No. 734: The Role of Transvaginal Ultrasonography in Evaluating the Endometrium of Women with Postmenopausal Bleeding. Obstet. Gynecol..

[65] Wolfman W. (2018). No. 249-Asymptomatic Endometrial Thickening. J. Obstet. Gynaecol. Can..

[66] Kim M.-J., Kim J.-J., Kim S.M. (2016). Endometrial Evaluation with Transvaginal Ultrasonography for the Screening of Endometrial Hyperplasia or Cancer in Premenopausal and Perimenopausal Women. Obstet. Gynecol. Sci..

[67] Piróg M., Kacalska-Janssen O., Bereza T., Jach R. (2019). The Thin Red Line-Postmenopausal Abnormal Uterine Bleeding with Endometrial Thickness Less than 4 mm. Contemp. Oncol..

[68] Long B., Clarke M.A., Morillo A.D.M., Wentzensen N., Bakkum-Gamez J.N. (2020). Ultrasound Detection of Endometrial Cancer in Women with Postmenopausal Bleeding: Systematic Review and Meta-Analysis. Gynecol. Oncol..

[69] Moradan S., Ghorbani R., Lotfi A. (2017). Agreement of Histopathological Findings of Uterine Curettage and Hysterectomy Specimens in Women with Abnormal Uterine Bleeding. Saudi Med. J..

[70] Clarke M.A., Long B.J., Morillo A.D.M., Arbyn M., Bakkum-Gamez J.N., Wentzensen N. (2018). Association of Endometrial Cancer Risk with Postmenopausal Bleeding in Women. JAMA Intern. Med..

[71] Kleebkaow P., Maneetab S., Somboonporn W., Seejornj K., Thinkhamrop J., Kamwilaisak R. (2008). Preoperative and Postoper-Ative Agreement of Histopathological Findings in Cases of Endometrial Hyperplasia. Asian Pac. J. Cancer Prev..

[72] Saygili H. (2006). Histopathologic Correlation of Dilatation and Currettage and Hysterectomy Specimens in Patients with Postmenopausal Bleeding. Eur. J. Gynaecol. Oncol..

[73] Kadirogullari P., Atalay C.R., Ozdemir O., Sari M.E. (2015). Prevalence of Co-Existing Endometrial Carcinoma in Patients with Pre-operative Diagnosis of Endometrial Hyperplasia. J. Clin. Diagn. Res..

[74] Epstein E., Ramirez A., Skoog L., Valentin L. (2001). Dilatation and Curettage Fails to Detect Most Focal Lesions in the Uterine Cavity in Women with Postmenopausal Bleeding. Acta Obstet. Gynecol. Scand..

[75] Lee D.O., Jung M.H., Kim H.Y. (2011). Prospective Comparison of Biopsy Results from Curettage and Hysteroscopy in Post-Menopausal Uterine Bleeding. J. Obstet. Gynaecol. Res..

[76] Barut A., Barut F., Arikan I., Harma M., Harma M.I., Ozmen B.U. (2012). Comparison of the Histopathological Diagnoses of Pre-operative Dilatation and Curettage and Hysterectomy Specimens. J. Obstet. Gynaecol. Res..

[77] NVOG (Dutch Society of Obstetrics and Gynaecology). http://nvogdocumenten.nl/uploaded/docs/definitief%20NVOG%20richtlijn%20PMB.pdf.

[78] (2011). Abnormal Vaginal Bleeding in Pre-, Peri- and Post-menopausal Women: A Diagnostic Guide for General Practitioners and Gynecologists. https://canceraustralia.gov.au/sites/default/files/publications/abnormal-vaginal-bleeding-pre-peri-and-post-menopausal-women-diagnostic-guide-general-practitioners/pdf/ncgc_a3_menopause_chart_june_2012_final.pdf.

[79] Chopra V., Sangha R., Gadde R., Alkhoory W. (2018). Endometrial Biopsies with Insufficient Tissue: Descriptive Analysis and Cancer Outcomes in Women Aged 50 and above [26G]. Obstet. Gynecol..

[80] Prendergast E.N., Misch E., Chou Y.-A., Roston A., Patel A. (2014). Insufficient Endometrial Biopsy Results in Women with Abnormal Uterine Bleeding. Obstet. Gynecol..

[81] Polyzos N.P., Mauri D., Tsioras S., Messini C., Valachis A., Messinis I.E. (2010). Intraperitoneal Dissemination of Endometrial Cancer Cells After Hysteroscopy. Int. J. Gynecol. Cancer.

[82] Gallos I.D., Alazzam M., Clark T.J. (2016). Management of Endometrial Hyperplasia. RCOG/BSGE Green-Top Guidel..

[83] Ofinran O., Balega J. (2016). The Value of Magnetic Resonance Imaging in Investigating Complex Atypical Hyperplasia of the Endometrium. Minerva Ginecol.

[84] Vetter M.H., Smith B., Benedict J., Hade E.M., Bixel K., Copeland L.J., Cohn D.E., Fowler J.M., O’Malley D., Salani R. (2019). Preoperative Predictors of Endometrial Cancer at Time of Hysterectomy for Endometrial Intraepithelial Neoplasia or Complex Atypical Hyperplasia. Am. J. Obstet. Gynecol..

[85] Matsuo K., Ramzan A., Gualtieri M.R., Mhawech-Fauceglia P., Machida H., Moeini A., Dancz C.E., Ueda Y., Roman L.D. (2015). Prediction of Concurrent Endometrial Carcinoma in Women with Endometrial Hyperplasia. Gynecol. Oncol..

[86] Lee N., Lee K., Kim K., Hong J.H., Yim G.W., Seong S.J., Lee B., Lee J., Cho J., Lim S. (2020). Risk of Occult Atypical Hyperplasia or Cancer in Women with Nonatypical Endometrial Hyperplasia. J. Obstet. Gynaecol. Res..

[87] Lacey J.V., Chia V.M., Rush B.B., Carreon D.J., Richesson U.A., Ioffe O.B., Ronnett B.M., Chatterjee N., Langholz B., Sherman M.E. (2012). Incidence Rates of Endometrial Hyperplasia, Endometrial Cancer and Hysterectomy from 1980 to 2003 within a Large Prepaid Health Plan. Int. J. Cancer.

[88] Hui L.S., Chin S.H.M., Goh C., Hui L.X., Mathur M., Kuei T.L.Y., Xian F.C.H. (2021). Non-Atypical Endometrial Hyperplasia: Risk Factors for Occult Endometrial Atypia and Malignancy in Patients Managed with Hysterectomy. Obstet. Gynecol. Sci..

[89] Gundem G., Sendag F., Kazandi M., Akercan F., Mgoyi L., Terek M.C. (2003). Preoperative and Postoperative Correlation of His-Topathological Findings in Cases of Endometrial Hyperplasia. Eur. J. Gynaecol. Oncol..

[90] Dolanbay M., Kutuk M.S., Uludag S., Bulut A.N., Ozgun M.T., Ozcelik B. (2015). Concurrent Endometrial Carcinoma in Hyster-Ectomy Specimens in Patients with Histopathological Diagnosis of Endometrial Hyperplasia in Curettage Specimens. Ginekol. Pol..

[91] Çakmak Y., Öge T., Uslu E., Kavak C.D., Aydin T.Ö. (2019). Evaluation of Concurrent Endometrial Cancer in Patients with Endo-Metrial Hyperplasia; 10 Years Experience as a Tertiary Center. Zeynep Kamil Tıp Bülteni.

[92] Chen Y.-L., Wang K.-L., Chen M.-Y., Yu M.-H., Wu C.-H., Ke Y.-M., Chen Y.-J., Chang Y.-Y., Hsu K.-F., Yen M.-S. (2013). Risk Factor Analysis of Coexisting Endometrial Carcinoma in Patients with Endometrial Hyperplasia: A Retrospective Observa-Tional Study of Taiwanese Gynecologic Oncology Group. J. Gynecol. Oncol..

[93] Travaglino A., Raffone A., Saccone G., D’Alessandro P., Arduino B., De Placido G., Mascolo M., Insabato L., Zullo F. (2019). Significant Risk of Occult Cancer in Complex Non-Atypical Endometrial Hyperplasia. Arch. Gynecol. Obstet..

[94] Averette H.E., Steren A., Nguyen H.N. (1993). Screening in Gynecologic Cancers. Cancer.

[95] Kimura T., Kamiura S., Yamamoto T., Seino-Noda H., Ohira H., Saji F. (2004). Abnormal Uterine Bleeding and Prognosis of Endometrial Cancer. Int. J. Gynecol. Obstet..

[96] Petru E., Sevelda P., Reinthaller A., Denison U., Wildt L., Lahousen M., Staudach A. Evaluation of the Endometrium in the Asymptomatic Patient. In Guideline of the Austrian Society of Gynecology and Obstetrics (OEGGG) and the Austrian Society of Gynecologic Oncology (AGO). Version October 2006. http://www.oeggg.at.

[97] Osmers R.G.W., Osmers M., Kühn W. (1995). Prognostic Value of Transvaginal Sonography in Asymptomatic Endometrial Cancers. Ultrasound Obstet. Gynecol..

[98] Desai V.B., Wright J.D., Gross C.P., Lin H., Boscoe F.P., Hutchison L.M., Schwartz P.E., Xu X. (2019). Prevalence, Characteristics, and Risk Factors of Occult Uterine Cancer in Presumed Benign Hysterectomy. Am. J. Obstet. Gynecol..

[99] Hartmann K.E., Fonnesbeck C., Surawicz T. (2019). Management of Uterine Fibroids.

[100] Pritts E.A., Vanness D.J., Berek J.S. (2015). The Prevalence of Occult Leiomyosarcoma at Surgery for Presumed Uterine Fibroids: A Meta-Analysis. Gynecol. Surg..

